# A policy brief: improving access and utilization of adolescent sexual and reproductive health services in Southern Ethiopia

**DOI:** 10.3389/fpubh.2024.1364058

**Published:** 2024-11-21

**Authors:** Negussie Boti Sidamo, Amene Abebe Kerbo, Kassa Daka Gidebo, Yohannes Dibaba Wado

**Affiliations:** ^1^School of Public Health, College of Medicine and Health Sciences, Arba Minch University, Arba Minch, Ethiopia; ^2^School of Public Health, College of Health Sciences and Medicine, Wolaita Sodo University, Wolaita Sodo, Ethiopia; ^3^African Population and Health Research Center, Nairobi, Kenya

**Keywords:** policy brief, adolescent sexual and reproductive health, health service utilization, barriers to healthcare access, policy recommendations, health system strengthening, Southern Ethiopia

## Abstract

**Background:**

Adolescent sexual and reproductive health (ASRH) is fundamental to the overall wellbeing and development of young people. In Southern Ethiopia, adolescents face significant barriers to accessing and utilizing ASRH services: they include limited information, pervasive cultural stigma, and inadequate healthcare infrastructure. Addressing these barriers is critical to reducing unintended pregnancies, preventing sexually transmitted infections, and achieving national and international health objectives. Despite the existence of policies aimed at improving ASRH, these challenges persist, highlighting the urgent need for targeted policy reforms and effective implementation strategies to ensure a healthier future for adolescents in the region.

**Main findings:**

Recent research has identified substantial barriers to ASRH services in Southern Ethiopia. Key challenges include widespread gaps in knowledge, socio-cultural stigma, and strong influences from family and peers, compounded by limited service availability. The absence of adolescent-friendly care, coupled with insufficient outreach efforts, further restricts access. Healthcare providers also encounter significant obstacles, such as stigma, inadequate training, and a lack of institutional support, which undermine their ability to deliver effective services. Service utilization varies markedly based on geographic location, economic status, and educational attainment, with approximately half of adolescents lacking a comprehensive understanding of sexual and reproductive health and rights (SRHR). Moreover, persistent stigma, low levels of social support, and limited self-efficacy continue to hinder the uptake of these essential services.

**Policy implications:**

To improve ASRH services in Southern Ethiopia, a multi-faceted approach is imperative. Increasing public education through school programs and awareness campaigns can mitigate stigma and misinformation, fostering a more supportive environment. Initiatives such as providing transportation support and establishing mobile health clinics will significantly improve access for adolescents in underserved areas. Strengthening healthcare services by offering specialized training for providers and establishing adolescent-friendly clinics will elevate the quality of care. Engaging families, communities, and peer networks is essential for building a supportive framework that encourages adolescents to utilize SRH services. Finally, implementing skill-building and counseling programs will empower adolescents to make informed decisions regarding their sexual and reproductive health, ultimately contributing to improved health outcomes across the region.

## Introduction

Adolescent sexual and reproductive health (ASRH) is crucial for the wellbeing and future prospects of young individuals, especially in Southern Ethiopia (1). Access to quality ASRH services significantly influences the health and development of adolescents in this region. However, many young people face substantial barriers that hinder their access to these essential services, resulting in alarmingly high rates of unintended pregnancies and sexually transmitted infections (STIs) (2, 3).

These barriers stem from a complex interplay of cultural taboos, economic constraints, and systemic deficiencies within the healthcare system (4–6). Cultural norms and stigma often render discussions about sexual and reproductive health taboo, leading to widespread misinformation and reluctance among adolescents to seek necessary care (3, 7). Economic factors, such as poverty and inadequate transportation, further restrict access to healthcare facilities (8). Compounding these issues is the scarcity of adolescent-friendly services and an inadequately trained workforce, which exacerbate low service utilization and contribute to poor health outcome (9, 10).

Addressing ASRH is not only vital for promoting health equity but also for advancing broader social and economic development goals (1, 11). Effective interventions must bridge the divide between national policies and local practices to improve health outcomes and strengthen community resilience (12). Despite the establishment of national frameworks, such as the Health Sector Transformation Plan and the National Reproductive Health Strategy, significant gaps remain in addressing the unique needs of adolescents (13, 14). These policies often overlook the specific barriers faced by this population and lack comprehensive strategies for effective implementation, a challenge further compounded by resource constraints and insufficient coordination among stakeholders (13, 15).

Research highlights the urgent need for targeted interventions due to high rates of unintended pregnancies and STIs among adolescents in Southern Ethiopia (16, 17). Data from the Ethiopian Demographic and Health Survey (EDHS) indicate that one in five girls becomes pregnant before the age of 18, leading to severe health risks and perpetuating cycles of poverty and limited educational opportunities (16). Similarly, a substantial proportion of adolescents are affected by STIs, underscoring the need for comprehensive and accessible SRH services (17, 18).

To effectively address these policy and implementation gaps, a holistic approach is required, including enhancements in public education, economic and logistical support, healthcare services, and adolescent empowerment (11). Bridging the gap between national policies and local practices is essential to improving health outcomes and promoting the wellbeing of both individuals and the broader community.

This policy brief outlines a strategic framework for implementing evidence-based recommendations aimed at improving ASRH services. By synthesizing current research and proposing actionable strategies, we aim to empower young people with access to comprehensive health services and accurate information. Our ultimate goal is to foster a healthier future for all youth in the region, enabling them to lead healthier and more productive lives. Through these targeted interventions, we can effectively address the barriers to ASRH services and promote the overall wellbeing of adolescents.

## Rationale and aim of the policy brief

Access to quality sexual and reproductive health (SRH) services for adolescents in Southern Ethiopia is a pressing issue that demands immediate intervention. Despite the recognized importance of these services in promoting adolescent health, significant barriers hinder both access and utilization. Sociocultural norms, stigma, and the lack of adolescent-friendly services critically impede access to essential SRH resources. Addressing these challenges is paramount to improving health outcomes and ensuring that SRH services are tailored to meet the specific needs of young people.

Adolescent sexual and reproductive health (ASRH) indicators in Southern Ethiopia are alarmingly low, with many adolescents lacking access to vital health services. Recent healthcare reforms have inadequately addressed the chronic underfunding and ineffective implementation of ASRH services, resulting in pronounced health disparities among the adolescent population (12, 19). Enhancing these services is essential for the wellbeing of adolescents, as healthier youth contribute to stronger communities and more resilient economies.

The aim of this policy brief is to advocate for the enhancement of ASRH services in Southern Ethiopia. To achieve this, we intend to identify and analyze the key barriers that impede access to these vital services in the region. By developing evidence-based policy recommendations, we aim to address these barriers effectively and facilitate the implementation of targeted interventions. Moreover, engaging stakeholders in constructive discussions will be pivotal to improving ASRH outcomes.

Through the successful attainment of these objectives, we seek to ensure that adolescents in Southern Ethiopia have comprehensive access to SRH services. This access is essential to improving health outcomes and empowering young individuals to lead healthier and more productive lives. By prioritizing these initiatives, we aspire to contribute positively to the overall wellbeing of the adolescent population in the region, ultimately fostering a healthier future for all.

This brief synthesizes recent research findings on ASRH services in Southern Ethiopia (7, 20–23), identifying critical challenges and offering actionable recommendations for policymakers, healthcare providers, and community stakeholders. Our objective is to advocate for a future in which every adolescent in Southern Ethiopia has the opportunity to lead a healthy and productive life.

Established in 2023 through the reorganization of the former Southern Nations, Nationalities, and Peoples’ Region (SNNPR), Southern Ethiopia is characterized by a rich diversity of ethnic groups, each with their unique cultural and socio-economic profiles (24). This focus on Southern Ethiopia underscores the urgent need for targeted policies and interventions to address significant health disparities, particularly with regard to ASRH. Historically, the region has faced considerable challenges related to healthcare access and outcomes, which have been exacerbated by the recent administrative transition.

Recognizing these urgent issues, this policy brief advocates for the development of targeted interventions and comprehensive programs designed to meet the unique needs of the adolescent population in Southern Ethiopia. By ensuring that every young person in this region has access to essential health services and accurate information, we aim to foster a healthier, more informed, and productive future for all adolescents.

## Methodology

To comprehensively investigate the barriers to adolescent sexual and reproductive health and identify effective interventions, we used a rigorous multi-method approach. Our research began with an evidence synthesis that systematically reviewed existing literature and reports on ASRH across sub-Saharan Africa (SSA) (7). This synthesis aimed to highlight key gaps, challenges, and best practices in service provision, ultimately generating actionable insights to inform policy recommendations. By integrating data from multiple sources, we contextualized our findings and provided evidence-based solutions to the barriers hindering ASRH in the region.

Following the evidence synthesis, we conducted primary qualitative research to identify context-specific barriers to ASRH services in our study area (23). This phase included in-depth interviews with 15 healthcare professionals who provide ASRH services to adolescents, focusing on operational and structural challenges such as resource constraints, inadequate training, and systemic delivery obstacles. These insights highlighted the complexities of service provision that impact adolescents’ access to essential health resources.

Subsequently, we executed a comprehensive community-based survey involving 1,172 participants. This survey was essential for quantifying service utilization and identifying family and community-level factors that influence adolescents’ access to SRH services (20, 22). Additionally, it provided valuable insights into adolescents’ knowledge of SRHR, informing effective strategies to improve service utilization (21). We used a stratified random sampling technique to ensure a representative sample, considering various demographics, such as age, gender, and socio-economic status. Our analytical framework comprised correlation studies to explore relationships between variables, multilevel mixed logistic regression models to identify key influencing factors, latent class analysis to assess population heterogeneity, and structural equation modeling to examine the mediating roles of self-efficacy and perceived social support. This robust methodological framework fosters a comprehensive understanding of the factors affecting adolescents’ engagement with SRH services, guiding targeted interventions and policy recommendations aligned with best practices in health research.

In addition to quantitative methodologies, we facilitated seven focus group discussions (FGDs) with 75 adolescents and conducted 10 key informant interviews with healthcare providers (9). The FGDs provided rich qualitative insights into the multi-faceted challenges adolescents face when accessing SRH services, allowing for a deeper exploration of their lived experiences. Conversely, the key informant interviews shed light on the systemic obstacles that healthcare providers encounter in delivering these essential services. By synthesizing these qualitative and quantitative approaches, we effectively captured both demand-side and supply-side barriers to SRH service provision. Ultimately, this comprehensive methodology, integrating evidence synthesis with original research, offers a holistic perspective on the barriers to and potential interventions for improving adolescent SRH services. This approach aims to cultivate a more effective and responsive health system that meets the unique needs of adolescents in Southern Ethiopia, thereby significantly contributing to the broader objectives of public health initiatives across the region.

## Compelling evidence

This section synthesizes key findings from recent research on ASRH services in Southern Ethiopia. Access to these services depends on factors such as availability, physical accessibility, affordability, and the adequacy of facilities and resources. Utilization, meanwhile, is influenced by individuals’ willingness and ability to seek and engage with these services. A review of six critical studies underscores the pressing need for policy reforms and targeted interventions to address these challenges effectively. The findings from these studies reveal several urgent issues:

**Barriers to access and utilization:** The study “Socio-Ecological Analysis of Barriers to Access and Utilization of Adolescent Sexual and Reproductive Health Services in Sub-Saharan Africa” identified a number of barriers affecting ASRH services, including individual knowledge gaps, family and peer influences, inadequate service availability, and socio-cultural stigma. To overcome these barriers, a multi-faceted approach that addresses each level of influence is essential (7).

**Barriers and Levesque’s framework:** The phenomenological study “Exploring Barriers to Accessing Adolescent Sexual and Reproductive Health Services in Southern Ethiopia” used Levesque’s framework to reveal complex barriers such as lack of awareness, cultural acceptance issues, and limited availability of adolescent-friendly services, financial constraints, and stigma. These barriers necessitate targeted interventions to improve provider training and service availability and to create a supportive environment for ASRH services (9).

**Ineffectiveness of existing policies implementation:** The study “Exploring Providers’ Perception toward the Provision of Sexual and Reproductive Health Services for Unmarried Adolescents in Gamo Zone” highlighted significant gaps in policy implementation, including inadequate outreach, poor provider training, and ineffective coordination. These issues, coupled with unsupportive health facility environments, point to the need for more effective policy enforcement and stakeholder collaboration to improve service delivery (23).

**Service utilization patterns:** The study “Adolescent Utilization of Sexual and Reproductive Health Services in Gamo Zone” revealed variability in service use influenced by access, health literacy, and social support. It indicated that interventions should be tailored to the specific needs of different demographic and socio-economic groups, with a focus on improving health literacy, fostering supportive networks, and strengthening service infrastructure (22).

**Role of stigma, social support, and self-efficacy:** The study “Sexual and Reproductive Health Services Utilization among Adolescents in Southern Ethiopia” emphasized that stigma and low social support reduce service utilization, while higher self-efficacy promotes it. Addressing stigma and strengthening social support networks are crucial to improving ASRH service uptake, along with initiatives to boost adolescents’ self-efficacy (20).

**SRHR knowledge gap:** The study highlighted that approximately half of adolescents lack a comprehensive understanding of sexual and reproductive health and rights (SRHR). This underscores the need for enhanced educational initiatives and the use of social media to disseminate accurate information. Additionally, improving job opportunities for adolescents can further support SRHR education and overall wellbeing (21).

## Strong arguments for policy reforms

1. **Economic and social benefits**: Investments in adolescent sexual and reproductive health (ASRH) services generate substantial economic and social returns. Improved ASRH outcomes contribute to higher educational attainment and expanded economic opportunities for young people, driving national development. Moreover, reducing adolescent pregnancies and STIs alleviates the financial burden on healthcare systems, enabling more efficient resource allocation (1, 15, 25). Prioritizing ASRH services is crucial for policymakers (11, 26). Comprehensive ASRH services, including family planning, sexual health education, and access to contraception, play a vital role in significantly reducing adolescent pregnancies and sexually transmitted infections (STIs) (11, 26). This not only reduces healthcare costs by minimizing the demand for expensive medical interventions but also enables a more efficient allocation of resources (27). Moreover, healthier adolescents are more likely to complete their education and participate in the workforce, thereby contributing to sustainable economic development (27). Addressing the ASRH needs of young people also fosters gender equality and empowers them to make informed health decisions, ultimately reducing poverty and improving societal wellbeing (28).
2. **Human rights perspective**: Access to comprehensive SRH services is a fundamental human right. Ensuring that adolescents have the necessary information and resources to make informed decisions about their health is crucial for upholding their rights and dignity. Comprehensive SRH education empowers young people to make autonomous choices, fostering healthier and more informed communities (21, 29). Reforming policies to support sexual and reproductive health (SRH) rights is essential to align national frameworks with international human rights standards that advocate for adolescents’ access to comprehensive SRH services (30, 31). Such reforms create a protective legal environment for young people’s health and wellbeing while promoting social equity by addressing barriers faced by marginalized populations, including those from low-income or rural backgrounds (30, 31). By prioritizing SRH rights, governments can ensure equal access to essential health services, improve health outcomes, foster social cohesion, and empower young individuals to engage actively in their communities (32).
3. **Evidence from successful programs**: Evidence from successful SRH programs in Ethiopia and similar contexts underscores the effectiveness of targeted interventions. Community-based initiatives that engage parents, religious leaders, and youth have proven successful in increasing SRH service utilization among adolescents (25, 33). Additionally, mobile health units and telehealth services have been demonstrated to be effective in reaching adolescents in remote areas, and offering timely and confidential SRH care (34). These successes underscore the significant potential of scaling up mobile health units and telehealth services to improve sexual and reproductive health (SRH) service delivery to underserved populations (35). Mobile health units provide critical on-the-ground support by delivering SRH education, counseling, and medical services directly to young individuals, thereby fostering trust and encouraging them to seek care (36). Concurrently, telehealth services offer virtual consultations and resources, addressing geographical and social barriers that may hinder access to traditional healthcare (37). By investing in these strategies, we can ensure that all adolescents receive the comprehensive and confidential SRH services they require, ultimately leading to improved health outcomes and enhanced community wellbeing (37).
4. **Addressing key barriers**: Research reveals multiple barriers to access and utilization of ASRH services, including gaps in knowledge, cultural stigma, inadequate service availability, and financial constraints. Effective policy reforms must address these barriers through a multi-faceted approach. Improving provider training, increasing the availability of adolescent-friendly services, and tackling socio-cultural stigma are critical steps. By implementing targeted interventions that address these specific challenges, policymakers can improve service accessibility and utilization, leading to better health outcomes for adolescents (9, 20, 22)
5. **Improving SRHR education**: The significant gap in SRHR knowledge among adolescents underscores the need for improved educational initiatives (38). Strengthening SRHR education programs with a rights-based approach and leveraging social media for broader outreach can significantly improve adolescents’ understanding of their health rights. Additionally, policies that support job opportunities for adolescents can further contribute to their SRHR education and overall wellbeing (21, 22). Investing in comprehensive education and information dissemination is essential to empowering adolescents and fostering healthier communities. By addressing topics such as sexual and reproductive health, mental health, nutrition, and life skills, young people acquire the knowledge necessary to make informed decisions regarding their wellbeing (39). Implementing effective strategies, including workshops, community outreach, and digital platforms, significantly improves access to vital information, particularly for those in remote or underserved areas (40). When adolescents are empowered through education, they are more likely to engage in positive health behaviors, seek appropriate healthcare services, and advocate for their peers, thereby promoting healthier choices and reducing the stigma surrounding health issues (39, 40).

## Evidence-based recommendations for policy reforms

This section outlines evidence-based recommendations for policy reforms aimed at improving access to and utilization of SRH services for adolescents. These recommendations draw on research findings and best practices identified by organizations such as the World Health Organization (WHO), UNICEF, and international non-governmental organizations (NGOs) such as Save the Children and Care. Integrating these references will provide readers with actionable insights and examples of effective interventions.

1. **Implementing multi-faceted interventions**: Research indicates that barriers to adolescent SRH service access include knowledge gaps, socio-cultural stigma, and inadequate service availability (7, 9). Policymakers should implement comprehensive interventions that focus on three key areas: enhancing provider training, improving service infrastructure, and addressing socio-cultural norms through community engagement and awareness campaigns. Multi-faceted strategies are essential to address the multiple challenges adolescents face in accessing and utilizing SRH services (23). For instance, the WHO recommends community-based awareness programs to educate adolescents about available services and reduce stigma (11).
2. **Strengthening policy enforcement and coordination:** Findings indicating gaps in policy implementation, outreach, and provider training (9) highlight the critical need for improved policy enforcement and improved coordination among stakeholders. To address these issues effectively, it is essential to incorporate supervision and training into existing frameworks, in addition to monitoring mechanisms. Policymakers should establish clear guidelines that not only define monitoring protocols but also emphasize the necessity of regular supervision. Effective supervision ensures that interventions are delivered with fidelity, maintaining adherence to established standards and practices. Additionally, comprehensive training programs for healthcare providers are vital to equip them with the necessary skills and knowledge to implement SRH policies effectively. Strengthening collaboration among government agencies, non-governmental organizations NGOs, and community leaders is imperative to improving the consistency and quality of SRH service delivery (9, 23). By fostering a coordinated approach that prioritizes supervision and training, we can significantly improve the impact of policy enforcement and achieve better health outcomes for the community (41).
3. **Tailoring interventions to demographic needs:** Research shows variability in SRH service utilization based on demographic and socio-economic factors (22), indicating the necessity for tailored interventions that address the specific needs of different adolescent groups. Improving health literacy, fostering supportive social networks, and improving service infrastructure are crucial to increasing service utilization. Targeted educational programs and community support initiatives can help bridge these gaps and improve overall service uptake (22). Best practices from organizations such as Save the Children emphasize the importance of context-specific adaptations in program design (42).
4. **Addressing stigma and strengthening social support**: The role of stigma and low social support in hindering SRH service utilization (20) highlights the need for initiatives aimed at combating stigma and strengthening social support. Policies should promote stigma reduction through public awareness campaigns and incorporate support mechanisms, such as counseling and peer support programs, to empower adolescents and encourage service utilization (20). Evidence from UNICEF programs demonstrates that peer support initiatives can significantly enhance young people’s willingness to seek services (43).
5. **Improving SRHR education and utilizing digital platforms:** The identified knowledge gap on SRHR among adolescents (21) necessitates robust educational initiatives. Policymakers should advocate for comprehensive SRHR education programs and leverage digital platforms to disseminate information widely. Integrating SRHR education into school curricula and utilizing mobile and online platforms can enhance accessibility and engagement. Additionally, policies that create job opportunities for adolescents can further support SRHR education and overall wellbeing (21). The WHO recommends the use of digital health tools as essential for improving education and access to healthcare resources. By leveraging technologies such as mobile applications, online platforms, and telemedicine, healthcare providers can deliver timely, relevant information, increasing awareness of available services (44). These tools facilitate interactive learning experiences that are particularly appealing to adolescents and young adults, and bridge geographical barriers for individuals in remote areas (44). By promoting digital literacy alongside health education, the WHO empowers individuals to make informed health decisions, ultimately leading to improved health outcomes and equitable access to care, while also strengthening resilient health systems that can adapt to evolving challenges (41).
6. **Investing in successful SRH programs:** Evidence from successful SRH programs in Ethiopia and comparable contexts (33, 34) demonstrates the effectiveness of community-based and mobile health initiatives. Policymakers should scale up these approaches, adapting successful strategies to local contexts. Community-based programs that engage parents, religious leaders, and youth, in conjunction with mobile health units and telehealth services, can significantly improve access to SRH services, particularly in remote areas (34). Leveraging insights from proven initiatives will provide a roadmap for policymakers seeking to improve adolescent SRH services. By implementing these recommendations, policymakers can significantly improve the effectiveness and accessibility of ASRH services, ultimately leading to better health outcomes and overall wellbeing for adolescents.

## Conclusion

Improving access to and utilization of ASRH services in Southern Ethiopia is crucial for the health and wellbeing of young people and the broader community. By addressing identified barriers and implementing targeted policy reforms, we can ensure that all adolescents have the opportunity to lead healthy and empowered lives. The proposed changes are not only necessary but also feasible, with the potential to yield significant benefits for individuals and society as a whole.

To effectively address barriers to ASRH service utilization, a multi-faceted approach is essential. This includes cultural, social, economic, and educational interventions to create a supportive environment for adolescents. Policymakers and stakeholders should focus on improving public education through school programs and awareness campaigns to reduce stigma and misinformation, fostering a supportive environment for young people. Addressing economic and logistical barriers by providing transportation support and mobile health clinics will ensure that adolescents in underserved areas can easily access services. Strengthening healthcare services through specialized training for providers and establishing adolescent-friendly clinics will improve the quality and accessibility of care. Engaging families, communities, and peer networks will create a supportive framework that encourages service use. Increasing adolescents’ self-efficacy with targeted skill-building and counseling programs will empower them to make informed decisions about their sexual and reproductive health.

By focusing on these areas, policymakers and stakeholders can significantly improve the health and wellbeing of adolescents in Southern Ethiopia. Implementing these strategies will address both systemic and personal barriers, leading to better health outcomes and a more equitable ASRH service system.
