# Supplementary material for: A policy brief: improving access and utilization of adolescent sexual and reproductive health services in Southern Ethiopia
Source: Front Public Health. 2024 Nov 21;12:1364058. doi: 10.3389/fpubh.2024.1364058 (PMC11617508; doi:10.3389/fpubh.2024.1364058)
Supplement: Supplementary file 1 [file Table_1.docx]

SUMMARY METHODS USED IN THE MAIN STUDIES

| **S.**  **No** | **Objectives** | **Study design** | **Study population** | | **Sample**  **size** | **Study Outcome/Research question** | **Sampling Strategies** | **Data**  **analysis** |
| --- | --- | --- | --- | --- | --- | --- | --- | --- |
| **01** | The objective this review was synthesizes qualitative literatures to understand adolescents' perspectives and experiences regarding barriers to accessing SRH services in SSA. | Systematic review | Those qualitative studies conducted in any country in SSA, and involving either male or female adolescents’ views. | | A total of 23 qualitative studies fulfilled the eligibility criteria | Actual and perceived barriers or challenges adolescents face in accessing SRH services. | The selection of studies was done Following PRISMA guidelines | A deductive approach was used to systematically summarize the finding using a socio-ecological framework to guide synthesis |
| **02** | To explore healthcare providers perception to provide SRH services for adolescents. | **Phenomenological study design** | All healthcare providers who were providing SRH services in selected health facilities. | | A total of 15 KIIs was conducted | How healthcare providers perceived the provision of SRH services for adolescents? | Purposive sampling | Thematic analysis |
| 03 | Current status of adolescent sexual and reproductive health services utilization and factors associated in Gamo Zone, Southern Ethiopia. | **Community based Cross Sectional study** | All adolescent aged 10 to 19 years old and live in study area. | | **1181** adolescents | SRH services utilization | A multi-stage stratified sampling technique | Descriptive analysis  Reliability analysis  Pearson correlation analyses  A logistic regression analysis  Meditational analysis |
| 04 | To explore barriers to accessing sexual and reproductive health services access for adolescents in Gamo Zone, Southern Ethiopia. | **Descriptive Phenomenological study design** | FGD | Adolescents | Seven FGD with 75 adolescents | What are the barriers accessing sexual and reproductive health services among adolescents? | Purposive sampling | Framework analysis approach using Levesque et al. Access to health care framework |
|  |  |  | KII | SRH services Providers | Ten KIIs |  |  |  |
| 05 | To explore preferences to accessing sexual and reproductive health service in Gamo Zone, Southern Ethiopia | **Descriptive Phenomenological study design** | FGD | Adolescents | Seven FGD with 75 adolescents | What are the preferences for adolescents to accessing SRH services? | Purposive sampling | Thematic analysis |
|  |  |  | KIIs | SRH services Providers | Ten KIIs |  |  |  |
